# Influenza Seasonality in the Tropics and Subtropics – When to Vaccinate?

**DOI:** 10.1371/journal.pone.0153003

**Published:** 2016-04-27

**Authors:** Siddhivinayak Hirve, Laura P. Newman, John Paget, Eduardo Azziz-Baumgartner, Julia Fitzner, Niranjan Bhat, Katelijn Vandemaele, Wenqing Zhang

**Affiliations:** 1 Global Influenza Program, World Health Organization, Geneva, Switzerland; 2 University of Washington, Seattle, Washington, United States of America; 3 Netherlands Institute for Health Services Research, Utrecht, The Netherlands; 4 Centers for Disease Control and Prevention, Atlanta, Georgia, United States of America; 5 Program for Appropriate Technology, Seattle, Washington, United States of America; Harvard School of Public Health, UNITED STATES

## Abstract

**Background:**

The timing of the biannual WHO influenza vaccine composition selection and production cycle has been historically directed to the influenza seasonality patterns in the temperate regions of the northern and southern hemispheres. Influenza activity, however, is poorly understood in the tropics with multiple peaks and identifiable year-round activity. The evidence-base needed to take informed decisions on vaccination timing and vaccine formulation is often lacking for the tropics and subtropics. This paper aims to assess influenza seasonality in the tropics and subtropics. It explores geographical grouping of countries into vaccination zones based on optimal timing of influenza vaccination.

**Methods:**

Influenza seasonality was assessed by different analytic approaches (weekly proportion of positive cases, time series analysis, etc.) using FluNet and national surveillance data. In case of discordance in the seasonality assessment, consensus was built through discussions with in-country experts. Countries with similar onset periods of their primary influenza season were grouped into geographical zones.

**Results:**

The number and period of peak activity was ascertained for 70 of the 138 countries in the tropics and subtropics. Thirty-seven countries had one and seventeen countries had two distinct peaks. Countries near the equator had secondary peaks or even identifiable year-round activity. The main influenza season in most of South America and Asia started between April and June. The start of the main season varied widely in Africa (October and December in northern Africa, April and June in Southern Africa and a mixed pattern in tropical Africa). Eight “influenza vaccination zones” (two each in America and Asia, and four in Africa and Middle East) were defined with recommendations for vaccination timing and vaccine formulation. The main limitation of our study is that FluNet and national surveillance data may lack the granularity to detect sub-national variability in seasonality patterns.

**Conclusion:**

Distinct influenza seasonality patterns, though complex, could be ascertained for most countries in the tropics and subtropics using national surveillance data. It may be possible to group countries into zones based on similar recommendations for vaccine timing and formulation.

## Introduction

Influenza disease affects up to 10% of the world’s population every year [1]. The World Health Assembly resolved in 2003 to increase the use of seasonal influenza vaccines to protect individuals at high risk for influenza and related complications [2]. The World Health Organization (WHO) Global Action Plan for Influenza Vaccines, launched in 2006, aims to promote demand and use of seasonal influenza vaccine as a strategy towards pandemic preparedness [3]. Seasonal influenza activity peaks coincide with the colder months (November–February and May–October) in the temperate regions of the northern and southern hemisphere respectively [4]. The WHO Global Influenza Surveillance and Response System reviews bi-annually the antigenic characteristics of influenza viruses circulating globally during the year to predict which variants are likely to predominate in the following influenza season. It then recommends the vaccine composition for the following influenza season in the Northern and Southern hemisphere, in February and September, respectively [5]. Vaccine production takes about 6–7 months. The Northern hemisphere formulation is available by October whereas the Southern hemisphere formulation is available by April of the following year [6].

The last decade has seen an increasing number of low and middle income countries in the tropics and subtropics introducing seasonal influenza vaccination in their immunization programs or expanding to include maternal influenza immunization, especially in the Latin America region [7–10]. The biannual vaccine composition–production cycle is timed suitably for the temperate regions with distinct influenza seasonality patterns. Seasonal influenza activity is less well defined and poorly understood in the tropics and subtropics with multiple peaks and identifiable year-round activity that frequently coincides with the rainy season [11,12]. Influenza surveillance and pandemic preparedness has improved in many countries in the tropics and subtropics in recent years and now provides the opportunity to ascertain seasonality in countries with less distinct patterns [13]. This paper reports on a collaborative effort to assess influenza seasonality in the tropics and subtropics using four different approaches. It further defines geographical grouping of countries into vaccination zones with the aim to propose simplified operational guidance to countries on when to vaccinate and which formulation to use.

## Methods

We brought together four major global analysis efforts conducted in 2014 that assessed global influenza seasonality by the Centers for Disease Control and Prevention (CDC), Netherlands Institute for Health Services Research (NIVEL), Program for Appropriate Technology (PATH), and the WHO. From these analyses, data were sought from 138 countries and territories (except Australia) situated wholly or partly between the 38^th^ lateral north and south that typically have a tropical and subtropical climate ecosystem. The groups used different statistical approaches to analyse laboratory confirmed influenza activity from the FluNet reporting system and / or national surveillance data (Table 1). FluNet was the largest global database for influenza virological surveillance maintained by the WHO Global Influenza Surveillance and Response System since 1995 [14]. The FluNet database provided virological and epidemiological information on clinical specimens routinely collected from patients consulting at sentinel hospitals and clinics and tested for influenza virus by the National Influenza Centres in a country. The FluNet database was contributed by 143 National Influenza Centres in 113 countries and accessed at http://www.who.int/influenza/gisrs_laboratory/flunet/en/. FluNet database for the seasons 2010 to 2015 was included for analysis. Many countries also conducted sentinel surveillance for influenza-like illness and severe acute respiratory illness with or without laboratory confirmation for influenza infection. National surveillance data for the seasons 2002 to 2014 was included for analysis wherever available. The influenza pandemic year 2009–2010 was excluded as the influenza activity in this period was not seasonal by definition. The criteria for including data varied across sites and ranged from at least 50 to 100 reported influenza cases and / or at least 20 consecutive weeks of reported data in a year. The WHO researchers used time series analysis using the R statistical package, decomposed into seasonal, trend and residual components for years with more than 100 influenza cases to define peak activity. Significant levels in the autocorrelation function were regarded as signs of seasonality. The other groups estimated the monthly proportions of positive cases amongst all positives or amongst all tested within the year as a proxy for influenza activity. The PATH researchers determined a monthly proportion of positive influenza cases out of all positive cases within a given year and considered a month to have increased influenza activity if the monthly proportion was ≥ 10% for two or more years between 2010–2014. The CDC researchers further used a binomial model factored on month and year of activity to predict the monthly influenza activity i.e.

$$(i.e.,\log\;\left(\frac{Proportion\;of\;all\;samples\;testing\;positive\;for\;influenza}{1-Proportion\;of\;all\;samples\;testing\;positive\;for\;influenza})\;=\;year+month\right)$$

CDC also quantified the weighted average proportion of samples testing positive for influenza as a sensitivity analysis [15]. Two months or more of consecutive influenza activity above the annual median was considered epidemic. Criteria for defining a peak, number of peaks or thresholds for increased influenza activity differed (Table 1).

**Table 1:** Data source, definitions and analytic approach to assess influenza seasonality.

	PATH	NIVEL	CDC	WHO
**Data source**	1) FluNet	1) FluNet, 2) National surveillance data	1) FluNet, 2) PAHO, 3) National surveillance data	1) FluNet
**Seasons analysed**	2010–2015, 131 countries	FluNet: 2010–2014, 131 countries. National surveillance data: 2000–2014, 18 countries	2002–2014, 16 countries of Central and South America	2010–2014, 131 countries
**Inclusion**	Laboratory confirmed data	Laboratory confirmed data	Laboratory confirmed data. Countries with more than 3 years of monthly seasonal data	Laboratory confirmed data
**Exclusion**	1) 2009–2010, 2) Countries that reported less than 50 influenza cases in a year were excluded for that year	1) 2009 (2009–2010 for NH), 2) Season with less than 10 specimens per week (FluNet), 3) Season with less than 50 influenza cases or less than 20 consecutive weeks of reported data (National surveillance data)	1) 2009–2010, 2) Monthly data from countries that reported less than 12 months of continuous data 3) Less than 10 samples tested each month	1) 2009–2010, 2) Year with less than 100 influenza positive cases
**Approach used**	Weekly case proportion i.e. weekly proportion of positive cases over all positive cases for influenza within that year. Then converted to monthly case proportion.	FluNet: Months with high, low and no influenza activity were identified by eyeballing each year in the FluNet database. This assessment was made by two persons working independently. The annual assessments were then summarised by taking the average level of influenza activity per month (no, low or high). National surveillance data: Data were pooled on a monthly basis and a case proportion (i.e. a monthly proportion of samples testing positive for influenza) was calculated [16].	Weekly case proportion i.e. weekly proportion of samples testing positive for influenza. Then converted to monthly case proportion [17]. Binomial model to predict the monthly influenza activity as a factor of historical monthly and yearly activity–log(p/(1-p)) = year + month p = prop. of monthly influenza positives / total no. of monthly sample for testing. Also analysed whether peak influenza percentage positivity occurred during similar months each year.	Time Series analysis. Missing values in time series replaced with either 0 or moving average (imputation). Time series plot (observed and imputed) to check if imputation makes sense. Autocorrelation function plot to display dependency between time points.
**Criteria to define peak / period of increased influenza activity**	Month with 10% or more of total yearly cases of influenza for two or more years between 2010 and 2015. Second set of increased influenza activity separated by 2 or more months of non-peak activity.	1) FluNet: Eye-balling by two persons independently. The periods of increase influenza activity were defined as months with high levels of activity. 2) National surveillance data: Peak defined as week with highest no. of cases. If highest no. reported in two or more weeks, peak defined as the central week of the 3-wk or 5-wk period with the highest no. of reported cases. Then counted the no. of times the peak occurred in each month of the year. The monthly proportion of samples testing positive for influenza was used to identify months which had high levels of influenza activity.	Predicted influenza activity exceeded the annual median proportion of positive cases for at least 2 consecutive months. Start of epidemic defined as the first month when activity exceeded and remained above annual median proportion. End of epidemic defined as the month when activity remained below the annual median proportion for at least one month.	Time -series analysis to define peak. Decomposition plot to decompose time series into seasonal, trend and residual components.
**Criteria to define year-round activity**	Eight or more months of increased flu activity, or 3 or more peaks of influenza activity each separated by at least 2 months	Influenza was on average identified each month of the year	Influenza was on average identified each month of the year	

As a first step, a common database was agreed upon to define the number of peaks and the start and end month of the primary and secondary peak for each country. Influenza seasonality (number of peaks, identifiable year-round activity and period of increased influenza activity) was assessed by four different analytic approaches for each country and compared with seasonality reported in published literature. We assessed each country to decide if any of the methods resulted in a date range that was an outlier from the other methods. Discordance in the assessment of the number of peaks or where the start of the season differed by more than one month using the different analytic approaches, was resolved through discussions amongst the researchers and in-country experts by combining both the raw output of our individual methods and our knowledge of the strength of the data to determine outliers. The number of peaks and period of increased influenza activity assessed by each method and the consensus pattern for each country is given in S1 Table. Countries were further categorized as those with one or two distinct peaks with and without identifiable year-round influenza activity.

In the next step, countries with onset period of their primary influenza activity in the same calendar quarter were grouped into geographically contiguous zones that would indicate similar guidelines for vaccination timing. A country with inadequate data was presumed to have similar seasonality pattern as its neighbour for whom information was available, and grouped in the same zone as the neighbour. In the context where a country with no data adjoined neighbours that belonged to different zones, the country was grouped with the neighbour that shared the same latitude [12,17,18].

## Results

Of the 138 countries and territories in the tropics and subtropics, 73 had functional national influenza surveillance systems that reported to the Global Influenza Surveillance and Response System between 2002 and 2015. Influenza seasonality was assessed in 70 countries and territories representing about 73% of the world’s population. Seasonality could not be ascertained in 22 of the 48 countries in Africa due to inadequate data. The seasonality patterns assessed by the four different analytic approaches were discordant for 29 countries (21%), mostly in Africa (58%).

**Fig 1:**
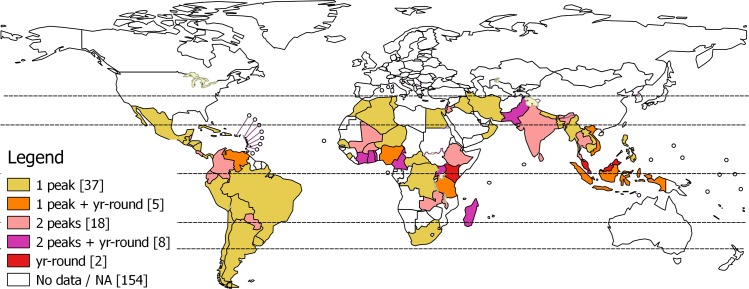
Influenza seasonality patterns—number of peaks and identifiable year-round activity. The number in parenthesis in legend indicate number of countries.

Most countries in Central and South America showed a single distinct peak except Belize, Colombia, Costa Rica, Ecuador, and Paraguay which showed two distinct peaks (Fig 1). Saharan Africa and countries in the Middle East showed a single distinct peak (exception–Jordan and Qatar which had two distinct peaks). Two distinct peaks were seen in Mali, Burkina Faso, Ethiopia, Mali, Rwanda. Furthermore, identifiable year-round activity was seen in countries situated nearer the equator (Côte d’Ivoire, Ghana, Togo, Nigeria, Cameroon, Kenya, Uganda, United Republic of Tanzania and Madagascar). Most countries in South and south-east Asia (Bangladesh, Cambodia, Lao People’s Democratic Republic, Myanmar, Nepal and Philippines) showed a single distinct peak (exception–Pakistan, India, Bhutan, Sri Lanka and Thailand showed two distinct peaks). Countries situated near the equator (Indonesia, Malaysia, Singapore and Viet Nam) showed identifiable year-round activity.

**Fig 2:**
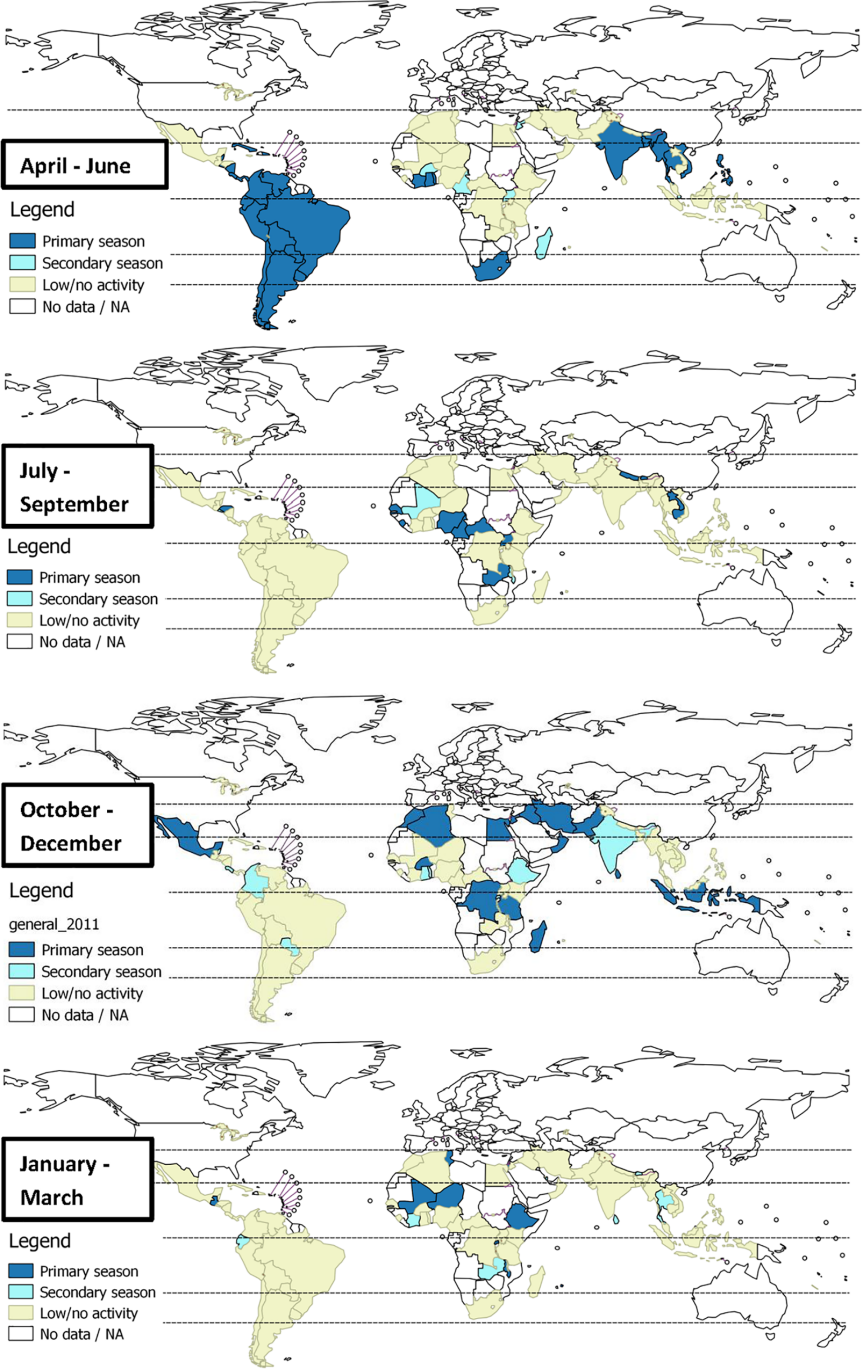
Primary and secondary influenza activity in the tropics and subtropics.

Fig 2 shows countries with primary or secondary periods of increased influenza activity in the four quarters of the calendar year. The primary period of influenza activity in most countries in Central and South America, South and Southeast Asia was from April to June. India showed an additional secondary peak between October and December. Africa presented a complex picture with increased activity from October to December in the northern region, from April to June in the southern region and throughout the year in sub-Saharan Africa near the equator.

**Fig 3:**
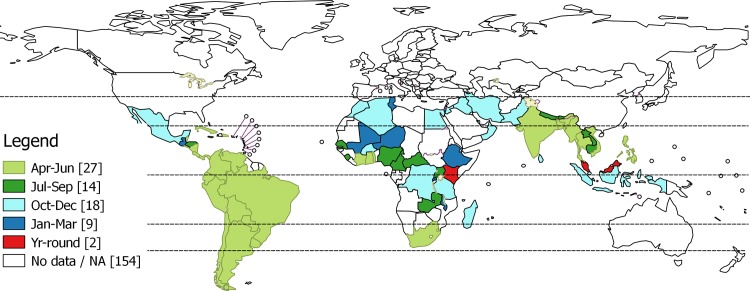
Start of the primary main influenza season. The number in parenthesis in legend indicate number of countries.

The start of the primary influenza season in most of tropical and subtropical America (except Mexico and Guatemala) and Asia (except Pakistan, Sri Lanka and Indonesia) was between April and June, similar to the seasonality pattern seen in the southern hemisphere temperate regions. The main season started a little later, between July and September, in Honduras and Jamaica in the Americas, and Nepal, Bhutan, Lao People’s Democratic Republic and Cambodia in Asia (Fig 3). The start of the main season varied in Africa. Most of Saharan Africa, the Middle East extending eastwards as far as Pakistan showed a seasonality pattern similar to the northern hemisphere temperate regions (start of the main influenza season between October and December). The primary season started between January and March in some countries of sub-Saharan Africa (Mali, Niger, Tunisia, Ethiopia, Rwanda, Malawi and Madagascar). West Africa (stretching from Senegal to the Central African Republic) and South Africa showed a seasonality pattern that was similar to that seen in the temperate regions of the southern hemisphere. The start of the primary influenza season was not ascertainable for Kenya and Malaysia (Table 2). Presuming that the southern and northern formulation become available in April and October respectively, the latest vaccine formulation would be available just prior to the onset of the primary influenza season for 45 out of 70 countries (47% of the world’s population) situated in the tropics and subtropics. In 23 countries (many of them in sub-Saharan Africa), there would be a 3 to 6 months lag between the onset of the primary influenza season and the availability of the existing vaccine formulation or the availability of the next updated vaccine formulation.

**Table 2:** Influenza seasonality assessment for Kenya, Malaysia.

	No. of peaks	Primary period of increased influenza activity	Secondary period of increased influenza activity	Seasons analysed	Data source
**Kenya**	**Year-round**	**Cannot be ascertained**			
CDC	2, year-round	Jul-Nov	Feb-Mar	2007–2013	ILI/SARI surveillance
NIVEL	1–2, year-round	Jan-Mar	Jul-Nov	2007–2013	FluNet & Surv data
PATH	Year-round			2010–2014	FluNet
WHO	Year-round			2010–2014	FluNet
Published [19,20]	1, year-round 3, year-round	Jul-Nov Mar-Apr & Oct-Nov	Jul	2007–2013	ILI/SARI surveillance
**Malaysia**	**Year-round**	**Cannot be ascertained**			
CDC					
NIVEL	1–2, year-round	Varied		2007–2010	FluNet
PATH	2	Jan-Feb	Apr-May	2010–2014	FluNet
WHO	Year-round	Inconclusive		2006–2008, 2010,2011,2014	FluNet
Published [18,21–23]	Year-round	May-Aug		2006–2011	ILI/SARI

(Note: Consensus finding in boldface)

**Fig 4:**
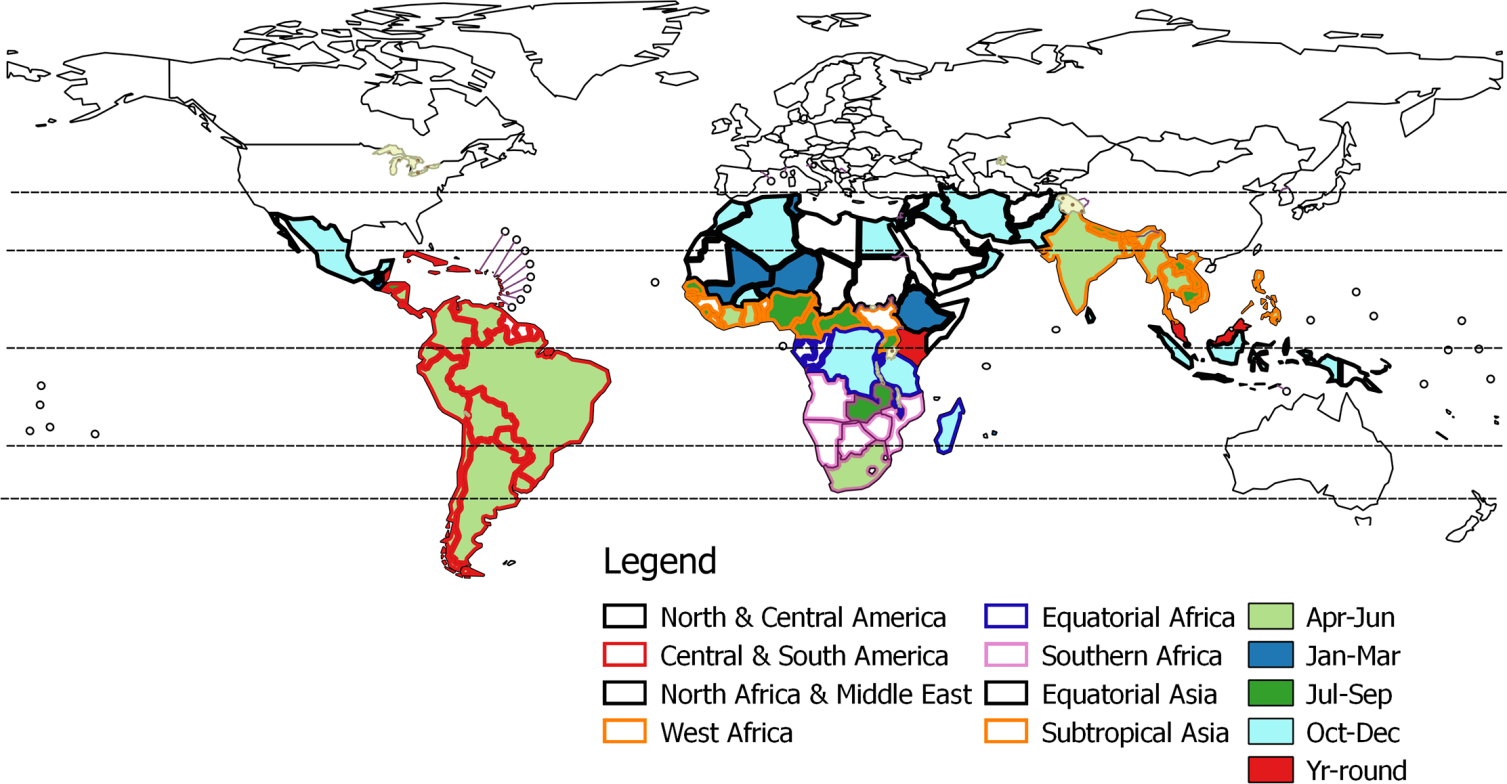
Seasonal influenza vaccination zones.

Countries with similar onset period of their main influenza season were grouped into eight geographically defined ‘influenza vaccination zones’ (Fig 4). Two zones each were proposed for America and Asia. A single zone was proposed for North Africa, the Middle East extending further eastwards to Pakistan. Separate zones were proposed for Saharan, equatorial and southern region of Africa. A southern hemisphere formulation is recommended for most of America (except Guatemala, Jamaica and Mexico), sub-Saharan Africa (except DR Congo, Malawi, Rwanda and United Republic of Tanzania), and tropical Asia (except Sri Lanka and Indonesia) to be given in April (Table 3). A northern hemisphere formulation to be given in October is appropriate for north Africa and the Middle East up to Pakistan.

**Table 3:** Influenza vaccination zones. Countries with data extrapolated from neighbours are in italics.

Vaccination zone	Countries from tropics and subtropics	Vaccine formulation	Vaccination timing
North & Central America	Guatemala, Jamaica, Mexico	NH	October
Central & South America (except–Guatemala, Jamaica)	*Anguilla*, *Antigua and Barbuda*, Argentina, *Bahamas*, *Barbados*, Belize, Bolivia (Plurinational State of), Brazil, *Cayman Islands*, Chile, Colombia, Costa Rica, Cuba, *Dominica*, Dominican Republic, Ecuador, El Salvador, *French Guiana*, *Grenada*, *Guyana*, *Haiti*, Honduras, *Montserrat*, *Netherland Antilles*, Nicaragua, Panama, Paraguay, Peru, *Saint Kitts and Nevis*, *Saint Lucia*, *Saint Vincent and the Grenadines*, *Suriname*, *Trinidad and Tobago*, *Turks and Caicos Islands*, Uruguay, Venezuela (Bolivarian Republic of)	SH	April
North Africa & Middle East	*Algeria*, Burkina Faso, *Chad*, *Djibouti*, Egypt, *Eritrea*, Ethiopia, *Libya*, Mali, *Mauritania*, Morocco, Niger, *Somalia*, *Sudan*, Tunisia, *Afghanistan*, Bahrain, Iran (Islamic Republic of), Iraq, Israel, Jordan, *Kuwait*, *Lebanon*, Oman, Pakistan, Qatar, *Saudi Arabia*, *Syrian Arab Republic*, *United Arab Emirates*, *Yemen*	NH	October
West Africa	*Benin*, Cameroon, Central African Republic, Côte d'Ivoire, *Gambia*, Ghana, *Guinea*, *Guinea-Bissau*, *Liberia*, Nigeria, Senegal, Sierra Leone, Togo, Uganda	SH	April
Equatorial Africa (except–Kenya, Uganda)	*Burundi*, *Congo (the)*, Democratic Republic of the Congo, *Equatorial Guinea*, *Gabon*, Madagascar, Malawi, Rwanda, United Republic of Tanzania	NH	October
Southern Africa	*Angola*, *Mozambique*, *Namibia*, South Africa, Zambia, *Zimbabwe*	SH	April
Tropical Asia	Bangladesh, Bhutan, Cambodia, India, Lao PDR, Myanmar, Nepal, Philippines, Thailand, Viet Nam	SH	April
Equatorial Asia (except—Malaysia)	*Brunei Darussalam*, Indonesia, Singapore, Sri Lanka	NH	October

(Note: Kenya and Malaysia were not assigned to any vaccination zone; NH = Northern hemisphere; SH = Southern hemisphere)

## Discussion

Laboratory confirmed influenza surveillance data were analysed for 70 countries representing 73% of the world population living in the tropics and subtropics to assess influenza seasonality. Despite differences in the surveillance methods, definition of thresholds, data sources, and seasons analysed, the different statistical approaches were consistent in ascertaining the number of peaks, and periods of increased influenza activity for majority (41 out of 70) of the countries assessed. Though the timing of the peaks varied slightly from season to season and some countries had an atypical peak occasionally, distinct patterns of increased influenza activity could be ascertained for most countries in the tropics and subtropics [24]. As seen in other studies, countries situated near to the equator were likely to have multiple peaks with or without identifiable year-round activity [18]. A distinct seasonality pattern could not be ascertained for two out of the 70 countries assessed, viz. Kenya and Malaysia. Notwithstanding, evidence from Kenya suggests that either formulation would be effective as each covered 50% the isolated viruses in Kenya (unpublished work).

Though the timing of the biannual influenza vaccine selection–production cycle has worked well for the temperate regions of the northern and southern hemispheres, its applicability to the seasonality seen in the tropics and subtropics is thought to be uncertain [25,26]. Our findings suggests that the optimal timing for the annual seasonal influenza vaccination campaign based on the start of the main influenza activity period, could be identified for most countries in the tropics and subtropics. Countries such as Brazil, China and India that have subnational variability in their seasonality pattern and countries situated near the equator with significant influenza activity throughout the year may need to consider alternate strategies based on their local seasonality [27–30]. The past experience of Peru in managing two schedules of vaccine procurement, deployment and withdrawal within the same year has been exceedingly challenging from a regulatory and logistics perspective (personal communication–PAHO). Instead, countries should consider strategies to increase vaccination coverage using the most appropriate formulation.

The FluNet data and the national surveillance data on which the analyses are based have inherent limitations. Though the FluNet database is the largest and most widely used global database for influenza strain information, its primary purpose is virological surveillance though it may be used for assessing seasonality and disease burden [31]. These data may not always be nationally representative and may lack the granularity to detect sub-national variability in seasonality patterns. Moreover, the data depend on reporting by the national influenza centres and sometimes subject to under-reporting.

The influenza vaccination zone is similar in concept to the 18 influenza transmission zones defined by the WHO since the 2009 pandemic, to monitor and report on the transmission dynamics of influenza activity globally [32]. Unlike the influenza vaccination zone, the influenza transmission zone does not take into consideration the vaccination perspective though one would expect a large degree of concordance between the two. The identification of influenza vaccination zones are an attempt to propose simplified operational guidance to countries in the tropics and subtropics on when to vaccinate and which formulation to use.

The proposed influenza vaccination zones are subject to a few limitations. First, the zones especially in Sub-Saharan Africa may be re-defined in alternate ways as more national surveillance data to ascertain seasonality become available. Second, the assumptions and criteria for grouping countries with inadequate data with their neighbours are also open to debate. Third, this approach assumes that either the northern or southern hemisphere vaccine formulation is the most appropriate in terms of antigenic match of the vaccine virus with circulating influenza viruses in the tropics and subtropics. While this may not always be the case, current data support this assumption. Seasonal influenza viruses evolve antigenically onward to escape immune pressure in the population [33]. Antigenic mapping of A/H3N2 influenza virus isolated in Thailand between 2002 and 2006 showed no evidence that the antigenic evolution was any different from influenza viruses isolated elsewhere in the world. A similar analysis for A(H3N2), B-Victoria and B-Yamagata for 17 other countries in the tropics and subtropics, also showed no evidence for substantial differences from the global patterns of antigenic evolution [34]. Finally, lack of disaggregated sub-national data may mask local and regional variations in seasonality patterns. It is possible that nationally aggregated data for a country with two single but distinct peaks at the subnational level at different times of the year may lead to an incorrect assessment of seasonality for that country. In such situations, countries need to assess seasonality at the subnational level for decisions on vaccination timing and formulation to use.

Thus once the time of vaccination is determined by local seasonality, from an antigenic evolution perspective, the most recent WHO influenza virus vaccine recommendation should be used, independent of the geographic location of the country. To this extent, this paper proposes simplified operational guidance for influenza immunization to countries on when to vaccinate and which formulation to use.

## Supporting Information

S1 Table Influenza seasonality in the tropics and subtropics. The consensus seasonality pattern for each country is shown in the first row for each country.
(DOCX)
