# Supplementary material for: Influenza Seasonality in the Tropics and Subtropics – When to Vaccinate?
Source: PLoS One. 2016 Apr 27;11(4):e0153003. doi: 10.1371/journal.pone.0153003 (PMC4847850; doi:10.1371/journal.pone.0153003)
Supplement: S1 Table — The consensus seasonality pattern for each country is shown in the first row for each country. (DOCX) [file pone.0153003.s001.docx]

S1 Table: Influenza seasonality in the tropics and subtropics. Discordant seasonality findings are highlighted in yellow. The consensus seasonality pattern is shown in the first row of each country.

| **Country** | **No. of peaks** | **Influenza primary period** | **Influenza secondary period** | **Seasons analysed** | **Data source** |
| --- | --- | --- | --- | --- | --- |
| Algeria | 1 | Dec-Mar |  |  |  |
| CDC |  |  |  |  |  |
| NIVEL | 1 | Jan |  | 2009-2013 | FluNet |
| PATH | 1 | Dec-Mar |  | 2010-2014 | FluNet |
| WHO | 1 | Dec-Mar |  | 2009-2015 | FluNet |
| Published |  |  |  |  |  |
| Angola |  |  |  |  |  |
| CDC |  |  |  |  |  |
| NIVEL |  |  |  |  |  |
| PATH |  |  |  |  |  |
| WHO |  |  |  |  |  |
| Published |  |  |  |  |  |
| Benin |  |  |  |  |  |
| CDC |  |  |  |  |  |
| NIVEL |  |  |  |  |  |
| PATH |  |  |  |  |  |
| WHO |  |  |  |  |  |
| Published |  |  |  |  |  |
| Botswana |  |  |  |  |  |
| CDC |  |  |  |  |  |
| NIVEL |  |  |  |  |  |
| PATH |  |  |  |  |  |
| WHO |  |  |  |  |  |
| Published |  |  |  |  |  |
| Burkina Faso | 2 | Nov-Mar | Jun-Oct |  |  |
| CDC |  |  |  |  |  |
| NIVEL | 1 | Oct-Feb |  | 2011-2013 | FluNet |
| PATH | 1 | Feb-Mar |  | 2010-2014 | FluNet |
| WHO | 2 | Mar | Aug-Nov | 2013-2014 | FluNet |
| Published ([1](#_ENREF_1)) | 2 | Nov-Mar | Jun-Oct | 2010-2012 |  |
| Burundi |  |  |  |  |  |
| CDC |  |  |  |  |  |
| NIVEL |  |  |  |  |  |
| PATH |  |  |  |  |  |
| WHO |  |  |  |  |  |
| Published |  |  |  |  |  |
| **Cabo Verde** |  |  |  |  |  |
| CDC |  |  |  |  |  |
| NIVEL |  |  |  |  |  |
| PATH |  |  |  |  |  |
| WHO |  |  |  |  |  |
| Published |  |  |  |  |  |
| **Cameroon** | 1-2 Year-round | Sep-Dec | May-Jun |  |  |
| CDC |  |  |  |  |  |
| NIVEL | 1-2, Year-round | Oct-Nov | May-Jun | 2008-2013 | FluNet & Surv data |
| PATH | 1 | Sep-Dec | Jun | 2010-2014 | FluNet |
| WHO | 1-2 | Oct-Dec & Jan-Apr | May-Jun | 2009-2014 | FluNet |
| Published ([2-4](#_ENREF_2)) | 1-2, Year-round | Dec-Mar (2008) |  | 2008-2009 | ILI |
| **Central African Republic** | 1 | Aug-Sep |  |  |  |
| CDC |  |  |  |  |  |
| NIVEL | 1 | Aug-Sep |  | 2012-2013 | FluNet |
| PATH | 1 | Aug-Sep |  | 2010-2014 | FluNet |
| WHO | 1 | Aug-Sep |  | 2012-2013 | FluNet |
| Published |  |  |  |  |  |
| **Chad** |  |  |  |  |  |
| CDC |  |  |  |  |  |
| NIVEL |  |  |  |  |  |
| PATH |  |  |  |  |  |
| WHO |  |  |  |  |  |
| Published |  |  |  |  |  |
| **Congo** |  |  |  |  |  |
| CDC |  |  |  |  |  |
| NIVEL |  |  |  |  |  |
| PATH |  |  |  |  |  |
| WHO |  |  |  |  |  |
| Published |  |  |  |  |  |
| **Côte d'Ivoire** | 2 Year-round | Jun-Nov | Mar |  |  |
| CDC |  |  |  |  |  |
| NIVEL | 1-2, Year-round | Varied | Varied | 2011-2013 | FluNet & Surv data |
| PATH | 2 | Jun-Nov | Mar | 2010-2014 | FluNet |
| WHO |  |  |  |  |  |
| Published ([2](#_ENREF_2), [4](#_ENREF_4), [5](#_ENREF_5)) | 2, Year-round | May-Jun | Oct | 2007-2010 | ILI |
| **DR Congo** | 1 | Dec-May |  |  |  |
| CDC |  |  |  |  |  |
| NIVEL | 1, Year-round | Dec-Apr |  | 2010-2013 | FluNet |
| PATH | 1 | Dec-May |  | 2010-2014 | FluNet |
| WHO | inconclusive |  |  |  | FluNet |
| Published |  |  |  |  |  |
| **Djibouti** |  |  |  |  |  |
| CDC |  |  |  |  |  |
| NIVEL |  |  |  |  |  |
| PATH |  |  |  |  |  |
| WHO |  |  |  |  |  |
| Published |  |  |  |  |  |
| **Egypt** | 1 | Dec-Feb |  |  |  |
| CDC |  |  |  |  |  |
| NIVEL | 1-2 | Dec-Feb | Apr-May | 2011-2014 | FluNet |
| PATH | 1 | Nov-Jan |  | 2010-2014 | FluNet |
| WHO |  |  |  |  |  |
| Published |  |  |  |  |  |
| **Equatorial Guinea** |  |  |  |  |  |
| CDC |  |  |  |  |  |
| NIVEL |  |  |  |  |  |
| PATH |  |  |  |  |  |
| WHO |  |  |  |  |  |
| Published |  |  |  |  |  |
| **Eritrea** |  |  |  |  |  |
| CDC |  |  |  |  |  |
| NIVEL |  |  |  |  |  |
| PATH |  |  |  |  |  |
| WHO |  |  |  |  |  |
| Published |  |  |  |  |  |
| **Ethiopia** | 2 | Mar-Apr | Oct-Nov |  |  |
| CDC |  |  |  |  |  |
| NIVEL | 2, Year-round | Mar-Apr | Nov | 2012-2013 | FluNet |
| PATH | 2 | Mar-Apr | Oct-Nov | 2010-2014 | FluNet |
| WHO | 2 | Oct-Nov | Mar-Apr | 2012-2014 | FluNet |
| Published |  |  |  |  |  |
| **Gabon** |  |  |  |  |  |
| CDC |  |  |  |  |  |
| NIVEL |  |  |  |  |  |
| PATH |  |  |  |  |  |
| WHO |  |  |  |  |  |
| Published |  |  |  |  |  |
| **Gambia** |  |  |  |  |  |
| CDC |  |  |  |  |  |
| NIVEL |  |  |  |  |  |
| PATH |  |  |  |  |  |
| WHO |  |  |  |  |  |
| Published |  |  |  |  |  |
| **Ghana** | 1-2 Year-round | Jun-Jul | Oct-Nov |  |  |
| CDC |  |  |  |  |  |
| NIVEL | 1-2, Year-round | Jun-Jul | Nov | 2009-2013 | FluNet |
| PATH | Year-round |  |  | 2010-2014 | FluNet |
| WHO | 2 | Jun-Jul | Oct-Nov | 2010-2014 | FluNet |
| Published |  |  |  |  |  |
| **Guinea** |  |  |  |  |  |
| CDC |  |  |  |  |  |
| NIVEL |  |  |  |  |  |
| PATH |  |  |  |  |  |
| WHO |  |  |  |  |  |
| Published |  |  |  |  |  |
| **Guinea-Bissau** |  |  |  |  |  |
| CDC |  |  |  |  |  |
| NIVEL |  |  |  |  |  |
| PATH |  |  |  |  |  |
| WHO |  |  |  |  |  |
| Published |  |  |  |  |  |
| **Kenya** | Year-round | Inconclusive |  |  |  |
| CDC | 2, Year-round | Jul-Nov | Feb-Mar | 2007-2013 | ILI/SARI |
| NIVEL | 1-2, Year-round | Jan-Mar | Jul-Nov | 2007-2013 | FluNet & Surv data |
| PATH | Year-round |  |  | 2010-2014 | FluNet |
| WHO | Year-round |  |  | 2010-2014 | FluNet |
| Published ([6](#_ENREF_6), [7](#_ENREF_7)) | 1, Year-round 3, Year-round | Jul-Nov Mar-Apr & Oct-Nov | Jul | 2007-2013 | ILI/SARI |
| **Liberia** |  |  |  |  |  |
| CDC |  |  |  |  |  |
| NIVEL |  |  |  |  |  |
| PATH |  |  |  |  |  |
| WHO |  |  |  |  |  |
| Published |  |  |  |  |  |
| **Libya** |  |  |  |  |  |
| CDC |  |  |  |  |  |
| NIVEL |  |  |  |  |  |
| PATH |  |  |  |  |  |
| WHO |  |  |  |  |  |
| Published |  |  |  |  |  |
| **Madagascar** | 2 Year-round | Dec-Mar | Jun-Sep |  |  |
| CDC |  |  |  |  |  |
| NIVEL | 1-2, Year-round | Jan-Jul | Oct-Dec | 2006-2013 | FluNet & Surv data |
| PATH | 2 | Nov-Feb | May-Jul | 2010-2014 | FluNet |
| WHO | 1, Year-round | Jun |  |  |  |
| Published ([2](#_ENREF_2), [4](#_ENREF_4), [8-10](#_ENREF_8)) | 1-2, Year-round Year-round | Sep-Oct (2009) Jun-Jul | Mar-Apr (2009) | 1992-2009 |  |
| **Malawi** | 2 | Feb-Apr | Jul-Nov |  |  |
| CDC | 2 | Feb-Apr | Jul-Nov | 2011-2013 | Hospital |
| NIVEL |  |  |  |  |  |
| PATH |  |  |  |  |  |
| WHO |  |  |  |  |  |
| Published |  |  |  |  |  |
| **Mali** | 2 | Jan-Mar | Sep-Oct |  |  |
| CDC |  |  |  |  |  |
| NIVEL | 1 | Oct-Feb |  | 2010-2011 | FluNet |
| PATH | 2 | Jan-Mar | Sep-Oct | 2010-2014 | FluNet |
| WHO | 1, 2 | Jan-Mar | Sep-Oct | 2010-2014 | FluNet |
| Published |  |  |  |  |  |
| **Mauritania** |  |  |  |  |  |
| CDC |  |  |  |  |  |
| NIVEL |  |  |  |  |  |
| PATH |  |  |  |  |  |
| WHO |  |  |  |  |  |
| Published |  |  |  |  |  |
| **Mauritius** | 1 | Mar-Jul |  |  |  |
| CDC |  |  |  |  |  |
| NIVEL |  |  |  |  |  |
| PATH | 1 | Mar-Jul |  | 2010-2014 | FluNet |
| WHO | Year-round |  |  | 2010-2014 | FluNet |
| Published |  |  |  |  |  |
| **Morocco** | 1 | Dec-Feb |  |  |  |
| CDC |  |  |  |  |  |
| NIVEL | 1 | Dec-Feb |  | 2010-2013 | FluNet & Surv data |
| PATH | 1 | Dec-Feb |  | 2010-2014 | FluNet |
| WHO | 1 | Dec-Feb |  | 2010-2014 | FluNet |
| Published ([4](#_ENREF_4), [11](#_ENREF_11)) | 1 | Oct-Apr |  | 1996-2009 | SARI |
| **Mozambique** |  |  |  |  |  |
| CDC |  |  |  |  |  |
| NIVEL |  |  |  |  |  |
| PATH |  |  |  |  |  |
| WHO |  |  |  |  |  |
| Published |  |  |  |  |  |
| **Namibia** |  |  |  |  |  |
| CDC |  |  |  |  |  |
| NIVEL |  |  |  |  |  |
| PATH |  |  |  |  |  |
| WHO |  |  |  |  |  |
| Published |  |  |  |  |  |
| **Niger** | 1 | Jan-Apr |  |  |  |
| CDC |  |  |  |  |  |
| NIVEL | 1 | Jan-Apr |  | 2010-2011 | FluNet |
| PATH | 1 | Jan-Feb |  | 2010-2014 | FluNet |
| WHO | inconclusive |  |  |  |  |
| Published |  |  |  |  |  |
| **Nigeria** | 1 Year-round | Sep-Oct |  |  |  |
| CDC |  |  |  |  |  |
| NIVEL | 1, Year-round | Sep |  | 2011 | FluNet |
| PATH | Year-round |  |  | 2010-2014 | FluNet |
| WHO | 2 | Oct | Feb | 2010-2014 | FluNet |
| Published |  |  |  |  |  |
| **Rwanda** | 2 | Feb-Jun | Oct-Nov |  |  |
| CDC |  |  |  |  |  |
| NIVEL | 2 | Mar | May | 2010-2013 | FluNet |
| PATH | 1 | Feb-Jun |  | 2010-2014 | FluNet |
| WHO | 1 | Feb-Jun |  | 2010-2014 | FluNet |
| Published ([4](#_ENREF_4), [12](#_ENREF_12)) | 2 | Feb-Mar | Oct-Nov | 2008-2010 | ILI |
| **Senegal** | 1 | Sep-Nov |  |  |  |
| CDC |  |  |  |  |  |
| NIVEL | 1, Year-round | Sep-Oct |  | 2010-2013 | FluNet |
| PATH | 1 | Sep-Nov |  | 2010-2014 | FluNet |
| WHO | 1 | Sep-Nov |  | 2010-2014 | FluNet |
| Published ([2](#_ENREF_2), [4](#_ENREF_4), [13-15](#_ENREF_13)) | 1 | Jun/Jul-Sep/Oct |  | 1996-2013 |  |
| **Sierra Leone** | 1 | Aug-Oct |  |  |  |
| CDC |  |  |  |  |  |
| NIVEL | 1 | Sep |  | 2011 | FluNet |
| PATH | 1 | Aug-Oct |  | 2010-2014 | FluNet |
| WHO | 1? | Aug-Oct |  | 2011-2012 | FluNet |
| Published |  |  |  |  |  |
| **Somalia** |  |  |  |  |  |
| CDC |  |  |  |  |  |
| NIVEL |  |  |  |  |  |
| PATH |  |  |  |  |  |
| WHO |  |  |  |  |  |
| Published |  |  |  |  |  |
| **South Africa** | 1 | May-Sep |  |  |  |
| CDC |  |  |  |  |  |
| NIVEL | 1 | Jun-Sep |  | 2006-2013 | FluNet & Surv data |
| PATH | 1 | May-Sep |  | 2010-2014 | FluNet |
| WHO | 1-2 | May-Jun | Oct | 1999-2014 | FluNet |
| Published ([4](#_ENREF_4), [16](#_ENREF_16), [17](#_ENREF_17)) | 1 | Jun-Aug |  | 1984-2008 | ILI |
| **Sudan** |  |  |  |  |  |
| CDC |  |  |  |  |  |
| NIVEL |  |  |  |  |  |
| PATH |  |  |  |  |  |
| WHO |  |  |  |  |  |
| Published |  |  |  |  |  |
| **Togo** | 2 Year-round | Apr-Jun | Oct-Jan |  |  |
| CDC |  |  |  |  |  |
| NIVEL | 1, Year-round | May |  | 2011 | FluNet |
| PATH | 1 | Oct-Jan |  | 2010-2014 | FluNet |
| WHO | Year-round | Dec |  |  |  |
| Published ([18](#_ENREF_18)) | 2 | Apr-Jun | Oct-Jan |  |  |
| **Tunisia** | 1 | Jan-Mar |  |  |  |
| CDC |  |  |  |  |  |
| NIVEL | 1 | Jan-Mar |  | 2008-2013 | FluNet |
| PATH | 1 | Jan-Mar |  | 2010-2014 | FluNet |
| WHO | 1 | Jan-Feb |  | 2005-2014 | FluNet |
| Published ([19](#_ENREF_19), [20](#_ENREF_20)) | 1 | Nov-Apr |  | 2008-2011 | ILI |
| **Uganda** | 2 Year-round | Aug-Nov | Apr-May |  |  |
| CDC |  |  |  |  |  |
| NIVEL | 1, Year-round | Oct |  | 2009-2013 | FluNet |
| PATH | 2 | Apr-May | Aug-Nov | 2010-2014 | FluNet |
| WHO | 2 | Oct-Nov | Feb-Mar | 2010-2014 | FluNet |
| Published ([21](#_ENREF_21)) | 1, Year-round | Jun-Nov |  | 2007-2010 | ILI/SARI |
| **United Republic of Tanzania** | 1 Year-round | Dec-Feb |  |  |  |
| CDC |  |  |  |  |  |
| NIVEL | 1, Year-round | Feb |  | 2009-2013 | FluNet |
| PATH | 2 | Dec-Feb | Apr | 2010-2014 | FluNet |
| WHO | 1 | Dec-Jan |  | 2010-2014 | FluNet |
| Published |  |  |  |  |  |
| **Zambia** | 2 | Jul-Oct | Mar-Apr |  |  |
| CDC |  |  |  |  |  |
| NIVEL | Year-round |  |  | 2010-2013 | FluNet |
| PATH | 2 | Jul-Oct | Mar | 2010-2014 | FluNet |
| WHO | 2 | Jul-Oct | Mar-Apr | 2010-2014 | FluNet |
| Published ([22](#_ENREF_22)) | 1 | Jun-Oct |  | 2008-2009 | ILI/SARI |
| **Zimbabwe** |  |  |  |  |  |
| CDC |  |  |  |  |  |
| NIVEL |  |  |  |  |  |
| PATH |  |  |  |  |  |
| WHO |  |  |  |  |  |
| Published |  |  |  |  |  |
| **Anguilla** |  |  |  |  |  |
| CDC |  |  |  |  |  |
| NIVEL |  |  |  |  |  |
| PATH |  |  |  |  |  |
| WHO |  |  |  |  |  |
| Published |  |  |  |  |  |
| **Antigua and Barbuda** |  |  |  |  |  |
| CDC |  |  |  |  |  |
| NIVEL |  |  |  |  |  |
| PATH |  |  |  |  |  |
| WHO |  |  |  |  |  |
| Published |  |  |  |  |  |
| **Argentina** | 1 | May-Oct |  |  |  |
| CDC | 1 | May-Oct |  | 1997-2013 | MOH |
| NIVEL | 1 | Jun-Oct |  | 2006-2013 | FluNet |
| PATH | 1 | Jun-Oct |  | 2010-2014 | FluNet |
| WHO | 1 | Jul-Aug |  | 2010-2014 | FluNet |
| Published ([23-26](#_ENREF_23)) | 1 1 | May-Sep Apr-Nov |  | 1994-2010 2010-2014 | ILI PAHO |
| **Bahamas** |  |  |  |  |  |
| CDC |  |  |  |  |  |
| NIVEL |  |  |  |  |  |
| PATH |  |  |  |  |  |
| WHO |  |  |  |  |  |
| Published |  |  |  |  |  |
| **Barbados** |  |  |  |  |  |
| CDC |  |  |  |  |  |
| NIVEL |  |  |  |  |  |
| PATH |  |  |  |  |  |
| WHO |  |  |  |  |  |
| Published |  |  |  |  |  |
| **Belize** | 2 | May-Aug | Dec-Jan |  |  |
| CDC | 2 | May-Aug | Dec-Jan | 2011-2013 | CAR |
| NIVEL |  |  |  |  |  |
| PATH |  |  |  |  |  |
| WHO |  |  |  |  |  |
| Published ([27](#_ENREF_27)) | 2, Year-round | Apr-Jun | Sep-Jan | 2010-2014 | PAHO |
| **Bolivia (Plurinational State of)** | 1 | May-Oct |  |  |  |
| CDC | 1 | May-Oct |  | 2011-2013 | PAHO |
| NIVEL | 1 | Jun-Sep |  | 2010-2013 | FluNet |
| PATH | 1 | May-Oct |  | 2010-2014 | FluNet |
| WHO | 2 | Jun-Aug | Nov-Feb | 2010-2014 | FluNet |
| Published ([27](#_ENREF_27)) | 1 | Apr-Nov |  | 2010-2014 | PAHO |
| **Brazil** | 1 | Apr-Aug |  |  |  |
| CDC | 1 | Mar-Jul |  | 2003-2008, 2011-2014 | FluNet, PAHO |
| NIVEL | 1 | Varied |  | 2010-2013 | FluNet |
| PATH | 1 | Apr-Aug |  | 2010-2014 | FluNet |
| WHO | 3 | Jun | Jan, Feb-Oct, Nov |  |  |
| Published ([28-33](#_ENREF_28)) | 1 | Mar-Apr (north Brazil) May-Jun (south Brazil) |  | 1999-2007 | ILI |
| **Cayman Islands** |  |  |  |  |  |
| CDC |  |  |  |  |  |
| NIVEL |  |  |  |  |  |
| PATH |  |  |  |  |  |
| WHO |  |  |  |  |  |
| Published |  |  |  |  |  |
| **Chile** | 1 | May-Sep |  |  |  |
| CDC | 1 | May-Sep |  |  |  |
| NIVEL | 1 | Apr-Aug |  | 2006-2013 | FluNet |
| PATH | 1 | May-Sep |  | 2010-2014 | FluNet |
| WHO | 1 | May-Sep |  | 2000-2014 | FluNet |
| Published ([27](#_ENREF_27)) | 1 | Apr/May-Nov |  | 2010-2014 | PAHO |
| **Colombia** | 2 | Apr-Jul | Dec-Jan |  |  |
| CDC | 2 | Apr-Jul | Dec-Jan | 2002-2007, 2011-2013 | FluNet, PAHO |
| NIVEL | 1, Year-round | Jun |  | 2010-2013 | FluNet |
| PATH | 2 | Apr-Jul | Jan | 2010-2014 | FluNet |
| WHO |  |  |  |  |  |
| Published ([27](#_ENREF_27)) | 1, Year-round | Mar-May |  | 2010-2014 | PAHO |
| **Costa Rica** | 2 | Jun-Nov | Dec-Jan |  |  |
| CDC | 2 | Jun-Sep | Nov-Dec | 2002-2008, 2011-2014 |  |
| NIVEL | 1 | Jun-Dec |  | 2010-2013 | FluNet & Surv data |
| PATH | 2 | Jun-Nov | Jan | 2010-2014 | FluNet |
| WHO | 2 | Jun-Sep | Dec-Jan | 2010-2014 | FluNet |
| Published ([27](#_ENREF_27)) | 1 | Sep-Oct | Dec-Jan | 2010-2014 | PAHO |
| **Cuba** | 1 | May-Sep |  |  |  |
| CDC | 1 | May-Sep |  | 2011-2014 | PAHO |
| NIVEL | 1 | Jul-Sep |  | 2010-2013 | FluNet |
| PATH | 1 | May-Sep |  | 2010-2014 | FluNet |
| WHO | 1-2 | May-Sep | Jan | 2010-2014 | 2010-2014 |
| Published |  |  |  |  |  |
| **Dominica** |  |  |  |  |  |
| CDC |  |  |  |  |  |
| NIVEL |  |  |  |  |  |
| PATH |  |  |  |  |  |
| WHO |  |  |  |  |  |
| Published |  |  |  |  |  |
| **Dominican Republic** | 1 | Apr-Jul |  |  |  |
| CDC | 2 | Jun-Jul | Sep-Dec | 2011-2014 | PAHO |
| NIVEL | 1 | May-Aug |  | 2010-2013 | FluNet |
| PATH | 1 | Apr-Jul |  | 2010-2014 | FluNet |
| WHO | 1 | Apr-Jul |  | 2010-2014 | FluNet |
| Published ([27](#_ENREF_27)) | 1-2 | Apr-Jul | Nov-Dec | 2010-2014 | PAHO |
| **Ecuador** | 2 | Jun-Sep | Jan-Mar |  |  |
| CDC | 2 | Jul-Sep | Dec-Feb | 2011-2014 | PAHO |
| NIVEL | 1-2 | Jul-Aug | Jan | 2010-2013 | FluNet |
| PATH | 2 | Jun-Sep | Jan-Mar | 2010-2014 | FluNet |
| WHO | 1-2 | Jun-Sep | Jan-Mar | 2009-2014 | FluNet |
| Published ([27](#_ENREF_27)) | 1-2 | May-Jul | Nov-Apr | 2010-2014 | PAHO |
| **El Salvador** | 2 | May-Jul | Sep-Oct |  |  |
| CDC | 1 | May-Sep |  | 2005-2008, 2011-2014 | MOH,  PAHO |
| NIVEL | 1 | Jun-Jul |  | 2010-2013 | FluNet |
| PATH | 2 | May-Jul | Sep | 2010-2014 | FluNet |
| WHO | 2 | May-Jul | Oct-Dec | 2010-2014 | FluNet |
| Published ([27](#_ENREF_27)) | 1-2, Year-round | Apr-Jun | Sep-Nov | 2010-2014 | PAHO |
| **Grenada** |  |  |  |  |  |
| CDC |  |  |  |  |  |
| NIVEL |  |  |  |  |  |
| PATH |  |  |  |  |  |
| WHO |  |  |  |  |  |
| Published |  |  |  |  |  |
| **Guatemala** | 1 | Jan-Aug |  |  |  |
| CDC | 1 | Jan-Aug | Oct-Nov | 2002, 2006-2008, 2011-2014 | MOH,  PAHO |
| NIVEL | 1 | Jan-Sep |  | 2010-2013 | FluNet & Surv data |
| PATH | 1 | Jan-Apr |  | 2010-2014 | FluNet |
| WHO | 1 | Jan- Aug |  | 2010-2014 | FluNet |
| Published |  |  |  |  |  |
| **Guyana** |  |  |  |  |  |
| CDC |  |  |  |  |  |
| NIVEL |  |  |  |  |  |
| PATH |  |  |  |  |  |
| WHO |  |  |  |  |  |
| Published |  |  |  |  |  |
| **Haiti** |  |  |  |  |  |
| CDC |  |  |  |  |  |
| NIVEL |  |  |  |  |  |
| PATH |  |  |  |  |  |
| WHO |  |  |  |  |  |
| Published |  |  |  |  |  |
| **Honduras** | 1 | Jul-Nov |  |  |  |
| CDC | 1 | Jul-Dec |  | 2008, 2011-2014 | MOH,  PAHO |
| NIVEL | 1 | Jul-Nov |  | 2010-2013 | FluNet & Surv data |
| PATH | 1 | Jul-Nov |  | 2010-2014 | FluNet |
| WHO | 1 | Jul-Nov |  | 2010-2014 | FluNet |
| Published ([27](#_ENREF_27)) | 1 | Jun-Oct |  | 2010-2014 | PAHO |
| **Jamaica** | 1 | Sep-Nov |  |  |  |
| CDC | 2 | Feb-Apr | Oct-Dec | 2011-2014 | PAHO |
| NIVEL | 1 | Sep-Nov |  | 2009-2013 | FluNet |
| PATH | 1 | Sep-Nov |  | 2010-2014 | FluNet |
| WHO | 1 | Sep-Nov |  | 2010-2014 | FluNet |
| Published |  |  |  |  |  |
| **Mexico** | 1 | Nov-Mar |  |  |  |
| CDC | 1 | Nov-Mar |  | 2011-2013 | NIC |
| NIVEL | 1 | Dec-Jan |  | 2010-2013 | FluNet |
| PATH | 1 | Dec-Mar |  | 2010-2014 | FluNet |
| WHO | 1 | Dec-Feb |  | 2010-2014 | FluNet |
| Published ([34](#_ENREF_34), [35](#_ENREF_35)) | 1 | Nov-Feb |  | 2010-2014 | PAHO |
| **Montserrat** |  |  |  |  |  |
| CDC |  |  |  |  |  |
| NIVEL |  |  |  |  |  |
| PATH |  |  |  |  |  |
| WHO |  |  |  |  |  |
| Published |  |  |  |  |  |
| **Netherlands Antilles** |  |  |  |  |  |
| CDC |  |  |  |  |  |
| NIVEL |  |  |  |  |  |
| PATH |  |  |  |  |  |
| WHO |  |  |  |  |  |
| Published |  |  |  |  |  |
| **Nicaragua** | 1 | Jun-Nov |  |  |  |
| CDC | 1 | Jun-Nov |  | 2005-2008, 2011-2014 | NIC,  PAHO |
| NIVEL | 1 | May-Oct |  | 2010-2013 | FluNet |
| PATH | 1 | Jul-Nov |  | 2010-2014 | FluNet |
| WHO |  |  |  |  |  |
| Published ([27](#_ENREF_27)) | 1 | May-Oct |  | 2010-2014 | PAHO |
| **Panama** | 1 | May-Sep |  |  |  |
| CDC | 1 | May-Sep |  | 2008, 2011-2014 | NIC,  PAHO |
| NIVEL | 1 | May-Sep |  | 2010-2013 | FluNet & Surv data |
| PATH | 1 | Jun-Jul |  | 2010-2014 | FluNet |
| WHO | 1 | Jun-Jul |  | 2007-2014 | FluNet |
| Published ([27](#_ENREF_27)) | 1 | May-Sep |  | 2010-2014 | PAHO |
| **Paraguay** | 2 | Jun-Aug | Dec-Jan |  |  |
| CDC | 2 | Jun-Aug | Nov-Jan | 2003-2005, 2011-2014 | NIC,  PAHO |
| NIVEL | 2 | Dec-Jan | Jun-Jul | 2010-2013 | FluNet |
| PATH | 2 | Jun-Aug | Dec | 2010-2014 | FluNet |
| WHO | 2 | Jun-Aug | Dec-Jan | 2010-2014 | FluNet |
| Published ([27](#_ENREF_27)) | 1-2 | May-Sep/Nov | Dec-Feb | 2010-2014 | PAHO |
| **Peru** | 1 | Jun-Oct |  |  |  |
| CDC | 1 | May-Oct |  | 2004-2008, 2011-2014 | FluNet |
| NIVEL | 1 | Jul-Sep |  | 2010-2013 | FluNet |
| PATH | 1 | Jun-Nov |  | 2010-2014 | FluNet |
| WHO | 2 | Jul/Aug | Jan/Feb | 2010-2014 | FluNet |
| Published ([36](#_ENREF_36)) | 1   1, Year-round | May-Jul (south highlands) Year-round (north coast) Jan-Apr (jungle) Aug-Sep |  | 2006-2008   2010-2014 | ILI   PAHO |
| **Saint Kitts and Nevis** |  |  |  |  |  |
| CDC |  |  |  |  |  |
| NIVEL |  |  |  |  |  |
| PATH |  |  |  |  |  |
| WHO |  |  |  |  |  |
| Published |  |  |  |  |  |
| **Saint Lucia** |  |  |  |  |  |
| CDC |  |  |  |  |  |
| NIVEL |  |  |  |  |  |
| PATH |  |  |  |  |  |
| WHO |  |  |  |  |  |
| Published |  |  |  |  |  |
| **Saint Vincent and the Grenadines** |  |  |  |  |  |
| CDC |  |  |  |  |  |
| NIVEL |  |  |  |  |  |
| PATH |  |  |  |  |  |
| WHO |  |  |  |  |  |
| Published |  |  |  |  |  |
| **Suriname** |  |  |  |  |  |
| CDC |  |  |  |  |  |
| NIVEL |  |  |  |  |  |
| PATH |  |  |  |  |  |
| WHO |  |  |  |  |  |
| Published |  |  |  |  |  |
| **Trinidad and Tobago** |  |  |  |  |  |
| CDC |  |  |  |  |  |
| NIVEL |  |  |  |  |  |
| PATH |  |  |  |  |  |
| WHO |  |  |  |  |  |
| Published |  |  |  |  |  |
| **Turks and Caicos Islands** |  |  |  |  |  |
| CDC |  |  |  |  |  |
| NIVEL |  |  |  |  |  |
| PATH |  |  |  |  |  |
| WHO |  |  |  |  |  |
| Published |  |  |  |  |  |
| **Uruguay** | 1 | Jun-Aug |  |  |  |
| CDC |  |  |  |  |  |
| NIVEL | 1 | May-Nov |  | 2007-2013 | FluNet |
| PATH | 1 | Jun-Aug |  | 2010-2014 | FluNet |
| WHO | 1 | Jun-Aug |  | 2010-2014 | FluNet |
| Published ([27](#_ENREF_27)) | 1 | Jul-Nov |  | 2010-2014 | PAHO |
| **Venezuela (Bolivarian Republic of)** | 1 Year-round | Jun |  |  |  |
| CDC | 1, Year-round | Jun |  |  | 2011 |
| NIVEL |  |  |  |  |  |
| PATH |  |  |  |  |  |
| WHO |  |  |  |  |  |
| Published ([37](#_ENREF_37)) | 1-2, Year-round | Jun (2007) Jan 2008 Jan & Oct (2009) Jun (2010) |  | 2006-2010 | ILI |
| **Afghanistan** |  |  |  |  |  |
| CDC |  |  |  |  |  |
| NIVEL |  |  |  |  |  |
| PATH |  |  |  |  |  |
| WHO |  |  |  |  |  |
| Published |  |  |  |  |  |
| **American Samoa** |  |  |  |  |  |
| CDC |  |  |  |  |  |
| NIVEL |  |  |  |  |  |
| PATH |  |  |  |  |  |
| WHO |  |  |  |  |  |
| Published |  |  |  |  |  |
| **Bangladesh** | 1 | May-Sep |  |  |  |
| CDC |  |  |  |  |  |
| NIVEL | 1 | Mar-Sep |  | 2010-2013 | FluNet |
| PATH | 1 | Apr-Sep |  | 2010-2014 | FluNet |
| WHO | 1-2 | Jun-Jul | Sep Oct | 2010-2014 | FluNet |
| Published ([38-40](#_ENREF_38)) | 1 | Jun-Sep May-Oct |  | 2008-2011 | ILI/SARI |
| **Bhutan** | 2 | Jul-Sep | Mar-Apr |  |  |
| CDC |  |  |  |  |  |
| NIVEL | 2 | Aug-Sep | Mar-Apr | 2011-2013 | FluNet & Surv data |
| PATH | 2 | Jul-Aug | Apr | 2010-2014 | FluNet |
| WHO | 2 | Jul-Aug | Mar-Apr | 2010-2014 | FluNet |
| Published |  |  |  |  |  |
| **Brunei Darussalam** |  |  |  |  |  |
| CDC |  |  |  |  |  |
| NIVEL |  |  |  |  |  |
| PATH |  |  |  |  |  |
| WHO |  |  |  |  |  |
| Published |  |  |  |  |  |
| **Cambodia** | 1 | Jul-Dec |  |  |  |
| CDC |  |  |  |  |  |
| NIVEL | 1 | Oct-Nov |  | 2008-2013 | FluNet |
| PATH | 1 | Sep-Dec |  | 2010-2014 | FluNet |
| WHO | 1 | Oct Nov |  | 2010-2014 | FluNet |
| Published ([41-44](#_ENREF_41)) | 1 | Jul-Dec |  | 2007-2011 | ILI |
| **China** | 1-2 | Jan (north provinces) Apr-Jun (south provinces) Mar & Oct (middle provinces) |  |  |  |
| CDC |  |  |  |  |  |
| NIVEL |  | Varied |  |  |  |
| PATH | 1 | Dec-Mar |  | 2010-2014 | FluNet |
| WHO | 1 | Dec-Mar |  | 2010-2014 | FluNet |
| Published ([45](#_ENREF_45)) | 1-2 | Jan (north provinces) Apr-Jun (south provinces) Mar & Oct (middle provinces) |  | 2005-2011 | ILI |
| **China - Hong Kong SAR** | 1-2 Year-round | Nov-Mar | May-Jul |  |  |
| CDC |  |  |  |  |  |
| NIVEL |  |  |  |  |  |
| PATH |  |  |  |  |  |
| WHO |  |  |  |  |  |
| Published ([46](#_ENREF_46), [47](#_ENREF_47)) | 1-2, Year-round | Nov-Mar | May-Jul | 1993-2013 | SARI/ILI |
| **China - Province of Taiwan** | 1-2 Year-round | Nov-Apr | Jun-Nov |  |  |
| CDC |  |  |  |  |  |
| NIVEL |  |  |  |  |  |
| PATH |  |  |  |  |  |
| WHO |  |  |  |  |  |
| Published ([48-50](#_ENREF_48)) | 1-2, Year-round | Nov-Apr | Jun-Nov | 2001-2006 | ILI |
| **India** | 2 | Jun-Aug | Dec-Feb |  |  |
| CDC |  |  |  |  |  |
| NIVEL | 1-2, Year-round | Jul-Sep | Mar | 2009-2013 | FluNet |
| PATH | 2 | Mar-Apr | Jun-Sep | 2010-2014 | FluNet |
| WHO | 2 | Jun-Aug | Feb-Mar | 2007-2014 | FluNet |
| Published ([38](#_ENREF_38), [51-53](#_ENREF_51)) | 2 | Jun-Aug | Dec-Feb | 2006-2011 |  |
| **Indonesia** | 1 Year-round | Nov-Apr |  |  |  |
| CDC |  |  |  |  |  |
| NIVEL | 1, Year-round | Oct-Feb |  | 2007-2013 | FluNet & Surv data |
| PATH | 1 | Nov-Apr |  | 2010-2014 | FluNet |
| WHO | 1 | Dec-Jan |  | 2010-2014 | FluNet |
| Published ([38](#_ENREF_38), [54](#_ENREF_54), [55](#_ENREF_55)) | Year-round | Dec-Jan |  | 2007-2011 | ILI/SARI |
| **Iran (Islamic Republic of)** | 1 | Dec-Feb |  |  |  |
| CDC |  |  |  |  |  |
| NIVEL | 1 | Dec-Apr |  | 2007-2013 | FluNet |
| PATH | 1 | Dec-Feb |  | 2010-2014 | FluNet |
| WHO | 1 | Dec-Feb |  | 2007-2014 | FluNet |
| Published ([56](#_ENREF_56), [57](#_ENREF_57)) | 1 | Nov-Apr |  | 1991-2001 |  |
| **Lao People's Democratic Republic** | 1 | Sep-Nov |  |  |  |
| CDC |  |  |  |  |  |
| NIVEL | 1 | Sep-Nov |  | 2011-2013 | FluNet |
| PATH | 1 | Sep-Nov |  | 2010-2014 | FluNet |
| WHO | 1 | Oct Nov |  | 2010-2014 | FluNet |
| Published ([38](#_ENREF_38), [41](#_ENREF_41), [58](#_ENREF_58)) | 1 | Aug-Dec |  | 2008-2011 | ILI |
| **Malaysia** | Year-round | Inconclusive |  |  |  |
| CDC |  |  |  |  |  |
| NIVEL | 1-2, Year-round | Varied |  | 2007-2010 | FluNet |
| PATH | 2 | Jan-Feb | Apr-May | 2010-2014 | FluNet |
| WHO | Year-round | Inconclusive |  | 2006-2008, 2010,2011,2014 | FluNet |
| Published ([38](#_ENREF_38), [41](#_ENREF_41), [59](#_ENREF_59), [60](#_ENREF_60)) | Year-round | May-Aug |  | 2006-2011 | ILI/SARI |
| **Maldives** |  |  |  |  |  |
| CDC |  |  |  |  |  |
| NIVEL |  |  |  |  |  |
| PATH |  |  |  |  |  |
| WHO |  |  |  |  |  |
| Published |  |  |  |  |  |
| **Myanmar** | 1 | Jun-Aug |  |  |  |
| CDC |  |  |  |  |  |
| NIVEL |  |  |  |  |  |
| PATH |  |  |  |  |  |
| WHO |  |  |  |  |  |
| Published ([61](#_ENREF_61)) | 1 | Jun-Aug |  | 2005-2007 | SARI |
| **Nepal** | 1 | Jul-Aug |  |  |  |
| CDC |  |  |  |  |  |
| NIVEL | 1 | Jul-Sep |  | 2011-2013 | FluNet |
| PATH | 1 | Jul-Aug |  | 2010-2014 | FluNet |
| WHO | 2 | Jul-Aug | Apr-May | 2011-2014 | FluNet |
| Published |  |  |  |  |  |
| **Pakistan** | 1-2 Year-round | Dec-Feb |  |  |  |
| CDC |  |  |  |  |  |
| NIVEL | 1 | Jan |  | 2009-2013 | FluNet |
| PATH |  |  |  |  |  |
| WHO | 2 | Dec-Jan | Jul | 2007-2014 | FluNet |
| Published ([62](#_ENREF_62)) | 1-2, Year-round | Year-round (flu B) Dec-Mar (A/H1) Aug-Oct (A/H3) |  | 2008-2011 | ILI/SARI |
| **Philippines** | 1 | Jun-Oct |  |  |  |
| CDC |  |  |  |  |  |
| NIVEL | 1, Year-round | Jul-Oct |  | 2006-2013 | FluNet |
| PATH | 1 | Jul-Oct |  | 2010-2014 | FluNet |
| WHO | inconclusive |  |  |  |  |
| Published ([38](#_ENREF_38), [41](#_ENREF_41)) | 1 | Jun-Oct |  | 2006-2011 | ILI |
| **Singapore** | 2 Year-round | Dec-Feb | Jun-Jul |  |  |
| CDC |  |  |  |  |  |
| NIVEL | 1-2, Year-round | Dec-Feb | varied | 2007-2013 | FluNet & Surv data |
| PATH | 2 | Dec-Feb | Jun-Jul | 2010-2014 | FluNet |
| WHO |  |  |  |  |  |
| Published ([38](#_ENREF_38), [41](#_ENREF_41), [63](#_ENREF_63), [64](#_ENREF_64)) | Year-round |  |  | 2007-2011 |  |
| **Sri Lanka** | 2 | Nov-Jan | Mar-Jun |  |  |
| CDC |  |  |  |  |  |
| NIVEL | 1, Year-round | Varied |  | 2009-2013 | FluNet |
| PATH | 2 | Nov-Jan | Mar-Jun | 2010-2014 | FluNet |
| WHO | 2 | Nov/Dec | Jun/Jul |  | FluNet |
| Published |  |  |  |  |  |
| **Thailand** | 2 | Jun-Nov | Jan-Mar |  |  |
| CDC |  |  |  |  |  |
| NIVEL | 1-2, Year-round | Jun-Nov | Jan-Feb | 2006-2013 | FluNet |
| PATH | 2 | Jul-Oct | Feb | 2010-2014 | FluNet |
| WHO | 1 | Oct-Jan |  | 2006-2014 | FluNet |
| Published ([38](#_ENREF_38), [65](#_ENREF_65), [66](#_ENREF_66)) | 2 | Jun-Aug Jun-Oct Jun-Nov | Oct-Feb Jan-Mar Jan-Mar | 2004-2010 2004-2005 2007-2011 | ILI/SARI ILI ILI |
| **Timor-Leste** |  |  |  |  |  |
| CDC |  |  |  |  |  |
| NIVEL |  |  |  |  |  |
| PATH |  |  |  |  |  |
| WHO |  |  |  |  |  |
| Published |  |  |  |  |  |
| **Viet Nam** | 1 Year-round | May-Sep |  |  |  |
| CDC |  |  |  |  |  |
| NIVEL | 1-2, Year-round | May-Sep |  | 2006-2013 | FluNet & Surv data |
| PATH | Year-round |  |  | 2010-2014 | FluNet |
| WHO | 2 |  |  |  |  |
| Published ([38](#_ENREF_38), [67](#_ENREF_67), [68](#_ENREF_68)) | 1, Year-round 2, Year-round 2, Year-round | May-Aug Jul-Sep (2008) Apr-Jun (2009) | Dec (2008) Sep-Dec (2009) | 2006-2011 2007-2010 2006-2007 | ILI/SARI |
| **Bahrain** | 1 | Nov-Feb |  |  |  |
| CDC |  |  |  |  |  |
| NIVEL |  |  |  |  |  |
| PATH | 1 | Nov-Feb |  | 2010-2014 | FluNet |
| WHO | inconclusive |  |  |  |  |
| Published |  |  |  |  |  |
| **Iraq** | 1 | Dec-Feb |  |  |  |
| CDC |  |  |  |  |  |
| NIVEL | 1 | Jan-Feb |  | 2011-2013 | FluNet |
| PATH | 1 | Jan-Feb |  | 2010-2014 | FluNet |
| WHO | 1 | Dec-Feb |  |  |  |
| Published |  |  |  |  |  |
| **Israel** | 1 | Jan-Mar |  |  |  |
| CDC |  |  |  |  |  |
| NIVEL | 1 | Jan-Feb |  | 2007-2013 | FluNet |
| PATH | 1 | Jan-Mar |  | 2010-2014 | FluNet |
| WHO | 1 | Jan-Mar |  | 2007-2014 | FluNet |
| Published ([69](#_ENREF_69)) | 1 | Jan | Nov | 1976-1977 | ILI |
| **Jordan** | 2 | Dec-Jan | Apr-May |  |  |
| CDC |  |  |  |  |  |
| NIVEL | 1 | Dec-Jan |  | 2010-2013 | FluNet |
| PATH | 2 | Dec-Jan | Apr | 2010-2014 | FluNet |
| WHO | inconclusive | Dec | May | 2010-2014 | FluNet |
| Published |  |  |  |  |  |
| **Kuwait** |  |  |  |  |  |
| CDC |  |  |  |  |  |
| NIVEL |  |  |  |  |  |
| PATH |  |  |  |  |  |
| WHO |  |  |  |  |  |
| Published |  |  |  |  |  |
| **Lebanon** |  |  |  |  |  |
| CDC |  |  |  |  |  |
| NIVEL |  |  |  |  |  |
| PATH |  |  |  |  |  |
| WHO |  |  |  |  |  |
| Published |  |  |  |  |  |
| **Oman** | 1 | Dec-Mar |  |  |  |
| CDC |  |  |  |  |  |
| NIVEL | 1 | Dec-Jan |  | 2010-2013 | FluNet |
| PATH | 1 | Dec-Mar |  | 2010-2014 | FluNet |
| WHO | 2 | Dec-Jan | Jul | 2010-2014 | FluNet |
| Published |  |  |  |  |  |
| **Qatar** | 2 | Nov-Jan | Mar-Apr |  |  |
| CDC |  |  |  |  |  |
| NIVEL | 1 | Dec-Mar |  | 2011-2013 | FluNet |
| PATH | 2 | Nov-Jan | Mar-Apr | 2010-2014 | FluNet |
| WHO | inconclusive | Dec | Apr | 2011-2014 | FluNet |
| Published |  |  |  |  |  |
| **Saudi Arabia** |  |  |  |  |  |
| CDC |  |  |  |  |  |
| NIVEL |  |  |  |  |  |
| PATH |  |  |  |  |  |
| WHO |  |  |  |  |  |
| Published |  |  |  |  |  |
| **Syrian Arab Republic** |  |  |  |  |  |
| CDC |  |  |  |  |  |
| NIVEL |  |  |  |  |  |
| PATH |  |  |  |  |  |
| WHO |  |  |  |  |  |
| Published |  |  |  |  |  |
| **United Arab Emirates** |  |  |  |  |  |
| CDC |  |  |  |  |  |
| NIVEL |  |  |  |  |  |
| PATH |  |  |  |  |  |
| WHO |  |  |  |  |  |
| Published |  |  |  |  |  |
| **Yemen** |  |  |  |  |  |
| CDC |  |  |  |  |  |
| NIVEL |  |  |  |  |  |
| PATH |  |  |  |  |  |
| WHO |  |  |  |  |  |
| Published |  |  |  |  |  |
| **Cook islands** |  |  |  |  |  |
| CDC |  |  |  |  |  |
| NIVEL |  |  |  |  |  |
| PATH |  |  |  |  |  |
| WHO |  |  |  |  |  |
| Published |  |  |  |  |  |
| **Democratic People's Republic of Korea** |  |  |  |  |  |
| CDC |  |  |  |  |  |
| NIVEL |  |  |  |  |  |
| PATH |  |  |  |  |  |
| WHO |  |  |  |  |  |
| Published |  |  |  |  |  |
| **Fiji** |  |  |  |  |  |
| CDC |  |  |  |  |  |
| NIVEL |  |  |  |  |  |
| PATH |  |  |  |  |  |
| WHO |  |  |  |  |  |
| Published |  |  |  |  |  |
| **French Polynesia** |  |  |  |  |  |
| CDC |  |  |  |  |  |
| NIVEL |  |  |  |  |  |
| PATH |  |  |  |  |  |
| WHO |  |  |  |  |  |
| Published |  |  |  |  |  |
| **Guam** |  |  |  |  |  |
| CDC |  |  |  |  |  |
| NIVEL |  |  |  |  |  |
| PATH |  |  |  |  |  |
| WHO |  |  |  |  |  |
| Published |  |  |  |  |  |
| **Kiribati** |  |  |  |  |  |
| CDC |  |  |  |  |  |
| NIVEL |  |  |  |  |  |
| PATH |  |  |  |  |  |
| WHO |  |  |  |  |  |
| Published |  |  |  |  |  |
| **Marshall islands** |  |  |  |  |  |
| CDC |  |  |  |  |  |
| NIVEL |  |  |  |  |  |
| PATH |  |  |  |  |  |
| WHO |  |  |  |  |  |
| Published |  |  |  |  |  |
| **Nauru** |  |  |  |  |  |
| CDC |  |  |  |  |  |
| NIVEL |  |  |  |  |  |
| PATH |  |  |  |  |  |
| WHO |  |  |  |  |  |
| Published |  |  |  |  |  |
| **New Caledonia** | 1 | Jul-Aug |  |  |  |
| CDC |  |  |  |  |  |
| NIVEL | 1 | Jul-Aug |  | 2009-2013 | FluNet |
| PATH |  |  |  |  |  |
| WHO |  |  |  |  |  |
| Published |  |  |  |  |  |
| **Niue** |  |  |  |  |  |
| CDC |  |  |  |  |  |
| NIVEL |  |  |  |  |  |
| PATH |  |  |  |  |  |
| WHO |  |  |  |  |  |
| Published |  |  |  |  |  |
| **Palau** |  |  |  |  |  |
| CDC |  |  |  |  |  |
| NIVEL |  |  |  |  |  |
| PATH |  |  |  |  |  |
| WHO |  |  |  |  |  |
| Published |  |  |  |  |  |
| **Papua New Guinea** |  |  |  |  |  |
| CDC |  |  |  |  |  |
| NIVEL |  |  |  |  |  |
| PATH |  |  |  |  |  |
| WHO |  |  |  |  |  |
| Published |  |  |  |  |  |
| **Samoa** |  |  |  |  |  |
| CDC |  |  |  |  |  |
| NIVEL |  |  |  |  |  |
| PATH |  |  |  |  |  |
| WHO |  |  |  |  |  |
| Published |  |  |  |  |  |
| **Solomon islands** |  |  |  |  |  |
| CDC |  |  |  |  |  |
| NIVEL |  |  |  |  |  |
| PATH |  |  |  |  |  |
| WHO |  |  |  |  |  |
| Published |  |  |  |  |  |
| **Tonga** |  |  |  |  |  |
| CDC |  |  |  |  |  |
| NIVEL |  |  |  |  |  |
| PATH |  |  |  |  |  |
| WHO |  |  |  |  |  |
| Published |  |  |  |  |  |
| **Vanuatu** |  |  |  |  |  |
| CDC |  |  |  |  |  |
| NIVEL |  |  |  |  |  |
| PATH |  |  |  |  |  |
| WHO |  |  |  |  |  |
| Published |  |  |  |  |  |

## References

1. Tarnagda Z, Yougbare I, Ilboudo AK, Kagone T, Sanou AM, Cisse A, et al. Sentinel surveillance of influenza in Burkina Faso: identification of circulating strains during 2010-2012. Influenza and other respiratory viruses. 2014;8(5):524-9.

2. Heraud JM, Njouom R, Rousset D, Kadjo H, Caro V, Ndiaye MN, et al. Spatiotemporal circulation of influenza viruses in 5 African countries during 2008-2009: a collaborative study of the Institut Pasteur International Network. The Journal of infectious diseases. 2012;206 Suppl 1:S5-13.

3. Njouom R, Mba SA, Noah DN, Gregory V, Collins P, Cappy P, et al. Circulation of human influenza viruses and emergence of Oseltamivir-resistant A(H1N1) viruses in Cameroon, Central Africa. BMC infectious diseases. 2010;10:56.

4. Radin JM, Katz MA, Tempia S, Talla Nzussouo N, Davis R, Duque J, et al. Influenza surveillance in 15 countries in Africa, 2006-2010. The Journal of infectious diseases. 2012;206 Suppl 1:S14-21.

5. Kadjo HA, Ekaza E, Coulibaly D, Kouassi DP, Nzussouo NT, Kouakou B, et al. Sentinel surveillance for influenza and other respiratory viruses in Cote d'Ivoire, 2003-2010. Influenza and other respiratory viruses. 2013;7(3):296-303.

6. Katz MA, Muthoka P, Emukule GO, Kalani R, Njuguna H, Waiboci LW, et al. Results from the first six years of national sentinel surveillance for influenza in Kenya, July 2007-June 2013. PloS one. 2014;9(6):e98615.

7. Matheka DM, Mokaya J, Maritim M. Overview of influenza virus infections in Kenya: past, present and future. The Pan African medical journal. 2013;14:138.

8. Razanajatovo NH, Richard V, Hoffmann J, Reynes JM, Razafitrimo GM, Randremanana RV, et al. Viral etiology of influenza-like illnesses in Antananarivo, Madagascar, July 2008 to June 2009. PloS one. 2011;6(3):e17579.

9. Soares JL, Ratsitorahina M, Rakoto Andrianarivelo M, Robinson R, Rousset D, Rasoazanamiarana LN, et al. [Epidemics of acute respiratory infections in Madagascar in 2002: from alert to confirmation]. Archives de l'Institut Pasteur de Madagascar. 2003;69(1-2):12-9.

10. Rabarijaona LP, Rakotondrarija NT, Rousset D, Soares JL, Mauclere P. [Influenza epidemiologic and virologic surveillance in Antananarivo from 1995 to 2002]. Archives de l'Institut Pasteur de Madagascar. 2003;69(1-2):20-6.

11. Barakat A, Ihazmad H, Benkaroum S, Cherkaoui I, Benmamoun A, Youbi M, et al. Influenza surveillance among outpatients and inpatients in Morocco, 1996-2009. PloS one. 2011;6(9):e24579.

12. Nyatanyi T, Nkunda R, Rukelibuga J, Palekar R, Muhimpundu MA, Kabeja A, et al. Influenza sentinel surveillance in Rwanda, 2008-2010. The Journal of infectious diseases. 2012;206 Suppl 1:S74-9.

13. Niang MN, Dosseh A, Ndiaye K, Sagna M, Gregory V, Goudiaby D, et al. Sentinel surveillance for influenza in Senegal, 1996-2009. The Journal of infectious diseases. 2012;206 Suppl 1:S129-35.

14. Dia N, Diene Sarr F, Thiam D, Faye Sarr T, Espie E, OmarBa I, et al. Influenza-like illnesses in Senegal: not only focus on influenza viruses. PloS one. 2014;9(3):e93227.

15. Dosseh A, Ndiaye K, Spiegel A, Sagna M, Mathiot C. Epidemiological and virological influenza survey in Dakar, Senegal: 1996-1998. The American journal of tropical medicine and hygiene. 2000;62(5):639-43.

16. McAnerney JM, Cohen C, Moyes J, Besselaar TG, Buys A, Schoub BD, et al. Twenty-five years of outpatient influenza surveillance in South Africa, 1984-2008. The Journal of infectious diseases. 2012;206 Suppl 1:S153-8.

17. Schoub BD, Gessner BD, Ampofo W, Cohen AL, Steffen CA. Afriflu2--second international workshop on influenza vaccination in the African continent--8 November 2012, Cape Town (South Africa). Vaccine. 2013;31(35):3461-6.

18. Maman I, Badziklou K, Landoh ED, Halatoko AW, Nzussouo TN, Defang GN, et al. Implementation of influenza-like illness sentinel surveillance in Togo. BMC public health. 2014;14:981.

19. El Moussi A, Pozo F, Ben Hadj Kacem MA, Ledesma J, Cuevas MT, Casas I, et al. Virological Surveillance of Influenza Viruses during the 2008-09, 2009-10 and 2010-11 Seasons in Tunisia. PloS one. 2013;8(9):e74064.

20. Lysaniuk B, Tabeaud M, Mejri W. Exploration of the link between climate and influenza in Tunisia: Preliminary study of the 2004-2005 epidemic season. Environnement, Risques et Sante. 2011;10(2):120-7.

21. Lutwama JJ, Bakamutumaho B, Kayiwa JT, Chiiza R, Namagambo B, Katz MA, et al. Clinic- and hospital-based sentinel influenza surveillance, Uganda 2007-2010. The Journal of infectious diseases. 2012;206 Suppl 1:S87-93.

22. Theo A, Liwewe M, Ndumba I, Mupila Z, Tambatamba B, Mutemba C, et al. Influenza surveillance in Zambia, 2008-2009. The Journal of infectious diseases. 2012;206 Suppl 1:S173-7.

23. Pontoriero AV, Baumeister EG, Campos AM, Savy VL, Lin YP, Hay A. Antigenic and genomic relation between human influenza viruses that circulated in Argentina in the period 1995-1999 and the corresponding vaccine components. Journal of clinical virology : the official publication of the Pan American Society for Clinical Virology. 2003;28(2):130-40.

24. Kusznierz G, Cociglio R, Beltramino JC, Pierini J, De Jorge J, Gomez A, et al. [Monitoring of activity of influenza in Santa Fe, Argentina, 2005-2010]. Revista chilena de infectologia : organo oficial de la Sociedad Chilena de Infectologia. 2014;31(2):131-8.

25. Savy VL, Baumeister EG, Pontoriero AV. [Antigenic relationship between influenza A (H3N2) strains circulating in Argentina and vaccine strains]. Medicina. 1999;59(3):225-30.

26. Uez OC, Knez V, Fernandez Pascua CA, Gutierrez ML, Sanchez-Pulido L, Valencia A, et al. Influenza virus epidemiological surveillance in Argentina, 1987-1993, with molecular characterization of 1990 and 1993 isolates. Revista panamericana de salud publica = Pan American journal of public health. 1998;4(6):405-10.

27. PAHO-WHO. Influenza and other Respiratory Viruses under Surveillance, 2010-2015 [Internet]. 2015. Available from: <http://ais.paho.org/phip/viz/ed_flu.asp>.

28. de Mello WA, de Paiva TM, Ishida MA, Benega MA, Dos Santos MC, Viboud C, et al. The dilemma of influenza vaccine recommendations when applied to the tropics: the Brazilian case examined under alternative scenarios. PloS one. 2009;4(4):e5095.

29. Freitas FT. Sentinel surveillance of influenza and other respiratory viruses, Brazil, 2000-2010. The Brazilian journal of infectious diseases : an official publication of the Brazilian Society of Infectious Diseases. 2013;17(1):62-8.

30. Moura FE, Perdigao AC, Siqueira MM. Seasonality of influenza in the tropics: a distinct pattern in northeastern Brazil. The American journal of tropical medicine and hygiene. 2009;81(1):180-3.

31. Alonso WJ, Viboud C, Simonsen L, Hirano EW, Daufenbach LZ, Miller MA. Seasonality of influenza in Brazil: a traveling wave from the Amazon to the subtropics. American journal of epidemiology. 2007;165(12):1434-42.

32. Motta FC, Siqueira MM, Lugon AK, Straliotto SM, Fernandes SB, Krawczuk MM. The reappearance of Victoria lineage influenza B virus in Brazil, antigenic and molecular analysis. Journal of clinical virology : the official publication of the Pan American Society for Clinical Virology. 2006;36(3):208-14.

33. Vidal LR, Siqueira MM, Nogueira MB, Raboni SM, Pereira LA, Takahashi GR, et al. The epidemiology and antigenic characterization of influenza viruses isolated in Curitiba, South Brazil. Memorias do Instituto Oswaldo Cruz. 2008;103(2):180-5.

34. Davila J, Chowell G, Borja-Aburto VH, Viboud C, Grajales Muniz C, Miller M. Substantial Morbidity and Mortality Associated with Pandemic A/H1N1 Influenza in Mexico, Winter 2013-2014: Gradual Age Shift and Severity. PLoS currents. 2014;6.

35. Noyola DE, Arteaga-Dominguez G. Contribution of respiratory syncytial virus, influenza and parainfluenza viruses to acute respiratory infections in San Luis Potosi, Mexico. The Pediatric infectious disease journal. 2005;24(12):1049-52.

36. Laguna-Torres VA, Gomez J, Ocana V, Aguilar P, Saldarriaga T, Chavez E, et al. Influenza-like illness sentinel surveillance in Peru. PloS one. 2009;4(7):e6118.

37. Comach G, Teneza-Mora N, Kochel TJ, Espino C, Sierra G, Camacho DE, et al. Sentinel surveillance of influenza-like illness in two hospitals in Maracay, Venezuela: 2006-2010. PloS one. 2012;7(9):e44511.

38. Saha S, Chadha M, Al Mamun A, Rahman M, Sturm-Ramirez K, Chittaganpitch M, et al. Influenza seasonality and vaccination timing in tropical and subtropical areas of southern and south-eastern Asia. Bulletin of the World Health Organization. 2014;92(5):318-30.

39. Azziz Baumgartner E, Dao CN, Nasreen S, Bhuiyan MU, Mah EMS, Al Mamun A, et al. Seasonality, timing, and climate drivers of influenza activity worldwide. The Journal of infectious diseases. 2012;206(6):838-46.

40. Zaman RU, Alamgir AS, Rahman M, Azziz-Baumgartner E, Gurley ES, Sharker MA, et al. Influenza in outpatient ILI case-patients in national hospital-based surveillance, Bangladesh, 2007-2008. PloS one. 2009;4(12):e8452.

41. Members of the Western Pacific Region Global Influenza Surveillance Response S, Dwyer D, Barr I, Hurt A, Kelso A, Reading P, et al. Seasonal influenza vaccine policies, recommendations and use in the World Health Organization's Western Pacific Region. Western Pacific surveillance and response journal : WPSAR. 2013;4(3):51-9.

42. Sreng B, Touch S, Sovann L, Heng S, Rathmony H, Huch C, et al. A description of influenza-like illness (ILI) sentinel surveillance in Cambodia, 2006-2008. The Southeast Asian journal of tropical medicine and public health. 2010;41(1):97-104.

43. Blair PJ, Wierzba TF, Touch S, Vonthanak S, Xu X, Garten RJ, et al. Influenza epidemiology and characterization of influenza viruses in patients seeking treatment for acute fever in Cambodia. Epidemiology and infection. 2010;138(2):199-209.

44. Mardy S, Ly S, Heng S, Vong S, Huch C, Nora C, et al. Influenza activity in Cambodia during 2006-2008. BMC infectious diseases. 2009;9:168.

45. Yu H, Alonso WJ, Feng L, Tan Y, Shu Y, Yang W, et al. Characterization of regional influenza seasonality patterns in China and implications for vaccination strategies: spatio-temporal modeling of surveillance data. PLoS medicine. 2013;10(11):e1001552.

46. Chiu SS, Lo JY, Chan KH, Chan EL, So LY, Wu P, et al. Population-based hospitalization burden of influenza a virus subtypes and antigenic drift variants in children in Hong Kong (2004-2011). PloS one. 2014;9(4):e92914.

47. Chan PK, Mok HY, Lee TC, Chu IM, Lam WY, Sung JJ. Seasonal influenza activity in Hong Kong and its association with meteorological variations. Journal of medical virology. 2009;81(10):1797-806.

48. Lee YM, Wang SF, Lee CM, Chen KH, Chan YJ, Liu WT, et al. Virological investigation of four outbreaks of influenza B reassortants in the northern region of Taiwan from October 2006 to February 2007. BMC research notes. 2009;2:86.

49. Jian JW, Chen GW, Lai CT, Hsu LC, Chen PJ, Kuo SH, et al. Genetic and epidemiological analysis of influenza virus epidemics in Taiwan during 2003 to 2006. Journal of clinical microbiology. 2008;46(4):1426-34.

50. Tsai HP, Wang HC, Kiang D, Huang SW, Kuo PH, Liu CC, et al. Increasing appearance of reassortant influenza B virus in Taiwan from 2002 to 2005. Journal of clinical microbiology. 2006;44(8):2705-13.

51. Chadha MS, Broor S, Gunasekaran P, Potdar VA, Krishnan A, Chawla-Sarkar M, et al. Multisite virological influenza surveillance in India: 2004-2008. Influenza and other respiratory viruses. 2012;6(3):196-203.

52. Roy S, Dahake R, Patil D, Tawde S, Mukherjee S, Athlekar S, et al. Characterization of influenza virus among influenza like illness cases in Mumbai, India. VirusDisease. 2014:1-4.

53. Vashishtha VM, Kalra A, Choudhury P. Influenza vaccination in India: position paper of Indian Academy of Pediatrics, 2013. Indian pediatrics. 2013;50(9):867-74.

54. Kosasih H, Roselinda, Nurhayati, Klimov A, Xiyan X, Lindstrom S, et al. Surveillance of influenza in Indonesia, 2003-2007. Influenza and other respiratory viruses. 2013;7(3):312-20.

55. Beckett CG, Kosasih H, Ma'roef C, Listiyaningsih E, Elyazar IR, Wuryadi S, et al. Influenza surveillance in Indonesia: 1999-2003. Clinical infectious diseases : an official publication of the Infectious Diseases Society of America. 2004;39(4):443-9.

56. Moattari A, Ashrafi H, Kadivar MR, Kheiri MT, Shahidi M, Arabpour M, et al. Antigenic variations of human influenza virus in Shiraz, Iran. Indian journal of medical microbiology. 2010;28(2):114-9.

57. Mokhtari-Azad T, Mohammadi H, Moosavi IA, Saadatmand Z, Nategh R. Influenza surveillance in the Islamic Republic of Iran from 1991 to 2001. Eastern Mediterranean health journal = La revue de sante de la Mediterranee orientale = al-Majallah al-sihhiyah li-sharq al-mutawassit. 2004;10(3):315-21.

58. Khamphaphongphane B, Ketmayoon P, Lewis HC, Phonekeo D, Sisouk T, Xayadeth S, et al. Epidemiological and virological characteristics of seasonal and pandemic influenza in Lao PDR, 2008-2010. Influenza and other respiratory viruses. 2013;7(3):304-11.

59. Khor CS, Sam IC, Chan YF. Epidemiology and seasonality of respiratory viral infections in hospitalised children in Kuala Lumpur, Malaysia. J University Malaya Medical Centre. 2013;16(Special):18.

60. Saat Z, Abdul Rashid TR, Yusof MA, Kassim FM, Thayan R, Kuen LS, et al. Seasonal influenza virus strains circulating in Malaysia from 2005 to 2009. The Southeast Asian journal of tropical medicine and public health. 2010;41(6):1368-73.

61. Dapat C, Saito R, Kyaw Y, Naito M, Hasegawa G, Suzuki Y, et al. Epidemiology of human influenza A and B viruses in Myanmar from 2005 to 2007. Intervirology. 2009;52(6):310-20.

62. Badar N, Bashir Aamir U, Mehmood MR, Nisar N, Alam MM, Kazi BM, et al. Influenza virus surveillance in Pakistan during 2008-2011. PloS one. 2013;8(11):e79959.

63. Doraisingham S, Goh KT, Ling AE, Yu M. Influenza surveillance in Singapore: 1972-86. Bulletin of the World Health Organization. 1988;66(1):57-63.

64. Chew FT, Doraisingham S, Ling AE, Kumarasinghe G, Lee BW. Seasonal trends of viral respiratory tract infections in the tropics. Epidemiology and infection. 1998;121(1):121-8.

65. Chittaganpitch M, Supawat K, Olsen SJ, Waicharoen S, Patthamadilok S, Yingyong T, et al. Influenza viruses in Thailand: 7 years of sentinel surveillance data, 2004-2010. Influenza and other respiratory viruses. 2012;6(4):276-83.

66. Waicharoen S, Thawatsupha P, Chittaganpitch M, Maneewong P, Thanadachakul T, Sawanpanyalert P. Influenza viruses circulating in Thailand in 2004 and 2005. Japanese journal of infectious diseases. 2008;61(4):321-3.

67. Horby P, Mai le Q, Fox A, Thai PQ, Thi Thu Yen N, Thanh le T, et al. The epidemiology of interpandemic and pandemic influenza in Vietnam, 2007-2010: the Ha Nam household cohort study I. American journal of epidemiology. 2012;175(10):1062-74.

68. Nguyen HT, Dharan NJ, Le MT, Nguyen NB, Nguyen CT, Hoang DV, et al. National influenza surveillance in Vietnam, 2006-2007. Vaccine. 2009;28(2):398-402.

69. Peled T, Weingarten M, Varsano N, Matalon A, Fuchs A, Hoffman RD, et al. Influenza surveillance during winter 1997-1998 in Israel. The Israel Medical Association journal : IMAJ. 2001;3(12):911-4.
